# Knowledge of Australia’s My Health Record and factors associated with opting out: Results from a national survey of the Australian general population and communities affected by HIV and sexually transmissible infections

**DOI:** 10.1371/journal.pdig.0000200

**Published:** 2023-03-01

**Authors:** Martin Holt, James MacGibbon, Anthony K. J. Smith, Timothy R. Broady, Mark D. M. Davis, Christy E. Newman

**Affiliations:** 1 Centre for Social Research in Health, UNSW Sydney, Sydney, Australia; 2 School of Social Sciences, Monash University, Melbourne, Australia; 3 Australian Human Rights Institute, UNSW Sydney, Sydney, Australia; Flinders University, AUSTRALIA

## Abstract

My Health Record is Australia’s national, digital, personal health record system. All Australians have a record in the system unless they choose to opt out of it. Concerns about privacy, security and unwanted sharing of data, particularly in marginalised populations, may impede its use. We conducted a national, online survey of Australians’ attitudes to digital health in April-June 2020. The sample (N = 2,240) was recruited from the general population and four priority populations affected by HIV and other sexually transmissible infections: gay and bisexual men, people living with HIV, sex workers, and trans and gender diverse people. This analysis assesses factors associated with greater knowledge of My Health Record and the likelihood of opting out of the system. Due to increased concerns about data privacy and misuse, we hypothesised that priority population members would know more about and be more likely to opt out of the system. We found that most of the sample (71.2%) knew little about My Health Record and 29.4% had opted out of the system. Greater knowledge of My Health Record was associated with younger age, having a university degree, having one or more health conditions, and being trans or gender diverse. Being a student, unemployed, receiving government benefits, or having poor self-reported health, were associated with less knowledge. Opting out of My Health Record was associated with having a university degree, one or more health conditions, and being a priority population member. The likelihood of opting out was lower among people born overseas, residents of Queensland, and people who were students, unemployed, or receiving government benefits. We recommend additional investment in community-based education to address people’s concerns about My Health Record and support people to use the system without compromising their health care, privacy, or security. Opting out may be a legitimate choice for people who perceive more risks than benefits from the system.

## Author summary

My Health Record is Australia’s national personal health record system. Concerns about data privacy, security, and misuse have affected engagement with the system. Members of communities affected by bloodborne viruses and sexually transmissible infections may have increased concerns about sharing personal health information due to stigma and discrimination. In 2020, we surveyed the Australian public and members of communities affected by HIV and sexually transmissible infections about their engagement with digital health systems like My Health Record. We found low levels of knowledge of My Health Record and over a quarter of the sample had opted out of the system. Those in more challenging circumstances (e.g. people who were unemployed, receiving government benefits, or who had poor self-reported health), knew less about My Health Record. Participants who had opted out of My Health Record were more likely to have a university degree, one or more health conditions, or to be a member of a community affected by HIV or sexually transmissible infections. These results are concerning, given that My Health Record is supposed to improve health care, particularly for people with chronic health conditions and members of marginalised communities. We recommend investment in community education to address concerns with the My Health Record system. Opting out or deleting one’s record is understandable for people who perceive more risks than benefits from the system.

## Introduction

The Australian Government introduced a national, personal health record system in 2012 [1]. The aim was for every Australian resident to have a secure digital record, collecting important medical information in one place, such as their treatment history, test results, and use of medication. According to the Australian Digital Health Agency [2], the intention was to facilitate more effective coordination of care for people with chronic and comorbid conditions, and give registered practitioners access to the same health information, thereby lessening the chance of errors in prescribing and treatment. Key features of the original system were that consumers would ‘opt in’ to having a record and would retain control over which health information was shared [3]. However, after low levels of uptake during the opt in period, the Australian Government decided to rename and relaunch the system as My Health Record in 2016 and changed it to an ‘opt out’ scheme in 2019 [4,5]. As of June 2022, there were 23.3 million records in the system [6], out of an estimated population of 25.9 million people [7].

Research conducted before and after the implementation of My Health Record suggests a range of issues that have affected rollout, similar to those observed in other countries with comparable personal health record systems [8,9]. Low levels of awareness and knowledge of the system were believed to have reduced the number of people opting in to My Health Record, as were concerns about privacy, security, and fears of unauthorised sharing or data misuse [1,3,10–12]. Men and older people were less likely to have created a record during the opt in phase [5]. Researchers suggested that My Health Record may be difficult to navigate for those with insufficient health literacy, with much of the consumer-facing information about the system requiring a high degree of literacy to comprehend it [11,13]. Trust in the government to maintain the system and keep the records secure has been identified as an issue [10,12,14], particularly for disadvantaged groups [3], who may perceive risks to privacy as more important than perceived benefits of sharing health information [15]. Public controversies about the capacity of federal government agencies to handle sensitive information occurred during the transition to the opt out scheme, such as the online version of the national Census temporarily failing, and the government issuing automated (and incorrect) debt recovery notices to welfare recipients [16]. Media coverage of this type of problem can heighten privacy concerns about sharing health information [17]. Even though most Australians now have a record, it is very unclear if knowledge of My Health Record and its potential benefits have improved. Very few record holders (less than one in a thousand) access their record each month, and most data in the system (like prescription information) is uploaded automatically [6,18]. Qualitative research found that even among engaged, female health consumers, awareness of My Health Record was low, accessing it could be difficult, and distrust of the federal government was a barrier to engagement [16].

In Australia, there are a range of priority populations recognised by the federal government as at increased risk of or affected by bloodborne viruses and sexually transmissible infections. These include gay and bisexual men, people who inject drugs, sex workers, transgender people, people living with hepatitis C or HIV, and Aboriginal and Torres Strait Islander people [19–23]. These populations may experience a range of health inequities, including greater difficulty finding and accessing appropriate health care, stigma and discrimination, and higher rates of comorbid conditions [24–27]. A personal, electronic health record could be of benefit to members of these priority populations, particularly if they have complex or specialist health needs, see multiple providers or are managing chronic conditions [28]. However, communities that experience stigma and discrimination (and particularly legal regulation or criminalisation) may be wary of sharing personal information in any form of health system record, and may hold additional concerns about digital health systems given their mechanisms for protecting privacy and security may be less well understood [14]. Some community organisations, particularly those representing people who inject drugs, sex workers, people living with HIV, and trans and gender diverse people, responded to the change to an ‘opt out’ system by creating educational resources and advocacy messages about the benefits and risks of My Health Record [29–35]. This suggests divergent influences on priority populations’ engagements with services like My Health Record.

We set out to assess levels of awareness and knowledge of My Health Record, and the likelihood of using or opting out of the service. In a departure from previous studies, we prioritised the inclusion of populations affected by bloodborne viruses (particularly HIV) and sexually transmissible infections, to identify any issues with the engagement of these communities. We tested the hypotheses that members of priority populations would i) know more about My Health Record than members of the general population and ii) be more likely to have opted out of My Health Record. More broadly, we sought to understand which groups might have concerns about the My Health Record system that needed to be addressed to increase participation in the system and realise its potential health benefits.

## Method

We conducted a national, online survey of Australian adults’ engagement with and attitudes to digital health in April-June 2020 [36]. Eligible participants were people aged 18 or over who lived in Australia. The survey was developed in partnership with a range of community organisations representing people affected by or at risk of HIV and sexually transmissible infections, including ACON, the Australian Federation of AIDS Organisations, LGBTIQ+ Health Australia, the National Association of People With HIV Australia, and Scarlet Alliance. Institutional ethics review and approval was obtained from the UNSW Human Research Ethics Committee (ref. HC191000), with additional appraisal from the community organisation ACON’s Research Ethics Review Committee (ref. RERC 201929).

Advertising and recruitment for the survey was conducted in several ways. The market research company Qualtrics Research Services was contracted to recruit a general population sample (target 1300–1500 people) from existing panels, with quotas for participants from every state and territory to achieve a representative sample in terms of state/territory of residence [37]. Participants received credits (which they could use towards an incentive like a gift card). To ensure the recruitment of priority populations, we worked with community organisations to actively promote the study through their social media channels and networks and placed paid advertising on Facebook. Facebook ads were aimed at Australian adults with four priority populations named and encouraged to participate: gay and bisexual men, people living with HIV, sex workers, and trans and gender diverse people. It was decided to limit the focus to four populations at risk of or affected by HIV and other sexually transmissible infections to ensure that recruitment could be completed in a timely manner with the resources that were available in the project. We were aware that this was likely to reduce participation of other groups named in the National Strategies e.g. people who inject drugs, people affected by viral hepatitis, and Aboriginal and Torres Strait Islander people. The aim was to recruit a minimum of 100 participants from each of the named priority populations. It should be noted that priority population members could be recruited through the Qualtrics panel recruitment, and general population members could respond to the ads directed at priority populations. All potential participants were directed to the survey website, shown the participant information, and asked to provide positive consent at the start of the survey (indicated by participants selecting the option ‘I agree/start survey’ after the statement, ‘I understand I am being asked to provide consent to participate in this research study’). Participants could participate anonymously, so names and contact details were not collected. Participants who responded to Facebook advertising or community organisation publicity could choose at the end of the survey to go into a draw to win one of twenty AU$50 gift cards.

The survey, which was hosted on UNSW’s Qualtrics (Provo, UT) account, assessed participants’ demographics (including membership of priority populations), levels of health and wellbeing, degree of access to the internet, use of online, mobile and digital health platforms, levels of trust in digital technologies and institutions, and experiences of stigma and discrimination [36]. Some of the items about trust in technologies and institutions were adapted from the Swinburne National Technology and Society Monitor [38]. A specific section of the survey covered knowledge and use of My Health Record and opting out or deleting the record. This section was preceded by the following statement: “This section asks questions about your thoughts and experiences of Electronic Health Records, including My Health Record. My Health Record is an online summary of your key health information.” Adaptive routing was used so that participants were only shown relevant questions, based on their previous answers.

The two outcome measures used in this analysis were knowledge of My Health Record and opting out or deleting My Health Record. Knowledge was assessed with the question, ‘How much do you know about My Health Record?” with answer options from ‘Know nothing about it” (1) to “Know a lot about it” (5). Participants who scored 4 or 5 on this question were classified as having more knowledge about My Health Record, while participants who scored 1–3 were classified as knowing less. Opting out or deleting My Health Record was determined from the question, ‘Have you ever accessed your My Health Record?’, with 6 answer options including ‘Yes, I have’ and ‘No, but I do have one’. Participants who responded ‘Yes, I have accessed my record but have since deleted it’ or ‘No, I opted out or deleted my record’ were classified as opting out or deleting their record. We chose to focus on opting out or deleting My Health Record (as opposed to active use of the record, for example) to identify people who may have heightened concerns about the system. Participants who had retained their records were asked what types of information in the My Health Record system were useful to them (from a list of 7 types) and the benefits of using the system (from 8 benefits) [36]. Participants who had opted out were asked to nominate why from a list of 11 reasons [36].

Multivariate logistic regression was employed to identify independent associations with each outcome (knowledge of My Health Record and opting out or deleting the record). Covariates included demographic details, socioeconomic status, having chronic health conditions, self-reported health status [39], receiving mental health care, and membership of the targeted priority populations. In terms of hypothesis testing, we anticipated that membership of some or all of the priority populations would be independently associated with greater knowledge of My Health Record and a higher likelihood of opting out or deleting the record, after controlling for the other covariates. Statistical assumptions and model diagnostics for logistic regression were assessed (none were violated), and there were no missing data for the model variables. Crude and adjusted odd ratios and 95% confidence intervals are reported. Reasons for opting out are also described, with bivariate analyses between priority population members and the general population made using Pearson’s chi-squared tests. Analyses were conducted using Stata version 16.1 (College Station, TX). The dataset used for this analysis is publicly available [40].

## Results

A total of 2,914 people started the online survey. After excluding partial, incomplete, duplicate or contradictory responses, 2,240 eligible participants were included in the final sample (a 76.9% completion rate). Most of the sample (n = 1,463, 65.3%) was recruited through the Qualtrics panel, the remainder (n = 777, 34.7%) through Facebook advertising and community organisation networks.

Participant characteristics are shown in Table 1. Participants ranged in age from 18 to 87 years, with a mean age of 42.5 years. Just over half the sample were women, 43.3% were men and 2.5% were non-binary people. Over three-quarters of the sample was born in Australia and just under a quarter was born overseas. A quarter of the sample had a high school level education, another quarter had a trade certificate, and just under half had a university degree. Over half the sample lived in the capital city of their state or territory. The majority of participants lived in the three most populous states (New South Wales, Victoria and Queensland), with just under a quarter of participants living in other jurisdictions. Under half the sample (43.9%) was in full-time employment or self-employed, 14.6% were employed part-time, 9.4% were students, and 27.7% were unemployed or receiving government benefits (not mutually exclusive categories). Over half the sample had an annual income of less than $80,000. Just under half the sample indicated they had one or more long-term health conditions, but over three-quarters of the sample rated their health as good, very good or excellent. Just over a quarter of the sample indicated they had received mental health care in the last year.

**Table 1:** Participant characteristics and factors associated with greater knowledge about My Health Record (N = 2,240).

	All	Less knowledge	More knowledge	Odds ratio (95% CI)	*p* value	Adjusted odds ratio (95% CI)	*p* value
	N = 2,240 (%)	n = 1,595 (%)	n = 645 (%)				
Mean age (SD)	42.5 (16.1)	43.1 (16.4)	41.0 (15.2)	0.99 (0.99–1.00)	0.006	0.99 (0.98–1.00)	**.013**
Gender							
Man/male	969 (43.3)	680 (42.6)	289 (44.8)	Ref		Ref	
Woman/female	1182 (52.8)	866 (54.3)	316 (49.0)	0.86 (0.71–1.04)	0.113	0.93 (0.74–1.17)	.551
Non-binary	56 (2.5)	31 (1.9)	25 (3.9)	1.90 (1.10–3.27)	0.021	0.81 (0.37–1.78)	.607
Other/Prefer not to answer	33 (1.5)	18 (1.1)	15 (2.3)	1.96 (0.97–3.94)	0.059	1.30 (0.56–2.99)	.538
Country of birth							
Australia	1699 (75.8)	1195 (74.9)	504 (78.1)	Ref			
Overseas	541 (24.2)	400 (25.1)	141 (21.9)	0.84 (0.67–1.04)	0.107		
Education level							
High school	602 (26.9)	482 (30.2)	120 (18.6)	Ref		Ref	
Trade certificate	584 (26.1)	439 (27.5)	145 (22.5)	1.33 (1.01–1.75)	0.043	1.27 (0.96–1.69)	.096
University degree	1054 (47.1)	674 (42.3)	380 (58.9)	2.26 (1.79–2.87)	<0.001	1.81 (1.40–2.33)	**< .001**
Residential location							
Capital city	1366 (61.0)	954 (59.8)	412 (63.9)	Ref			
Other city/regional, rural or remote area	874 (39.0)	641 (40.2)	233 (36.1)	0.84 (0.70–1.02)	0.074		
State or territory							
New South Wales	781 (34.9)	539 (33.8)	242 (37.5)	Ref		Ref	
Victoria	536 (23.9)	391 (24.5)	145 (22.5)	0.83 (0.65–1.05)	0.124	0.85 (0.66–1.10)	.224
Queensland	391 (17.5)	294 (18.4)	97 (15.0)	0.73 (0.56–0.97)	0.028	0.84 (0.63–1.12)	.24
Other jurisdictions	532 (23.8)	371 (23.3)	161 (25.0)	0.97 (0.76–1.23)	.78	1.08 (0.84–1.39)	.566
Employment status							
Full-time/self-employed	967 (43.2)	625 (39.2)	342 (53.0)	Ref		Ref	
Part-time	309 (13.8)	221 (13.9)	88 (13.6)	0.73 (0.55–0.96)	.026	0.86 (0.64–1.17)	.348
Student/unemployed/other	964 (43.0)	749 (47.0)	215 (33.3)	0.52 (0.43–0.64)	< .001	0.66 (0.51–0.86)	**.002**
Income level (AUD)							
Less than $40,000	859 (38.3)	657 (41.2)	202 (31.3)	Ref		Ref	Ref
$40,000−$79,999	650 (29.0)	461 (28.9)	189 (29.3)	1.33 (1.06–1.68)	.015	1.08 (0.83–1.42)	.573
$80,000−$120,000	376 (16.8)	238 (14.9)	138 (21.4)	1.89 (1.45–2.45)	< .001	1.24 (0.90–1.71)	.19
More than $120,000	188 (8.4)	115 (7.2)	73 (11.3)	2.06 (1.48–2.88)	< .001	1.25 (0.84–1.85)	.267
Prefer not to say	167 (7.5)	124 (7.8)	43 (6.7)	1.13 (0.77–1.65)	.536	1.04 (0.70–1.54)	.86
Health conditions							
No reported conditions	1166 (52.1)	887 (55.6)	279 (43.3)	Ref		Ref	
One or more long-term health conditions	1074 (47.9)	708 (44.4)	366 (56.7)	1.64 (1.37–1.98)	< .001	1.99 (1.61–2.47)	**< .001**
Self-assessed health status							
Good/very good/excellent	1730 (77.2)	1212 (76.0)	518 (80.3)	Ref		Ref	
Poor/fair	510 (22.8)	383 (24.0)	127 (19.7)	0.78 (0.62–0.97)	.027	0.73 (0.57–0.94)	**.015**
Receiving mental health care							
No	1602 (71.5)	1161 (72.8)	441 (68.4)	Ref		Ref	
Yes	638 (28.5)	434 (27.2)	204 (31.6)	1.24 (1.01–1.51)	.036	1.02 (0.81–1.29)	.859
Sexual identity							
Heterosexual	1622 (72.4)	1197 (75.0)	425 (65.9)	Ref		Ref	
Gay and bisexual men	277 (12.4)	175 (11.0)	102 (15.8)	1.64 (1.26–2.15)	0.551	1.11 (0.79–1.55)	0.551
Lesbian and bisexual women and other non-heterosexual participants	341 (15.2)	223 (14.0)	118 (18.3)	1.49 (1.16–1.91)	0.849	1.03 (0.75–1.41)	0.849
HIV status							
HIV-negative/Untested or unknown	2133 (95.2)	1529 (95.9)	604 (93.6)	Ref		Ref	
HIV-positive	107 (4.8)	66 (4.1)	41 (6.4)	1.57 (1.05–2.35)	.027	1.13 (0.71–1.79)	.62
Sex worker							
No	2101 (93.8)	1505 (94.4)	596 (92.4)	Ref			
Yes	139 (6.2)	90 (5.6)	49 (7.6)	1.37 (0.96–1.97)	.084		
Trans or gender diverse							
No	2110 (94.2)	1525 (95.6)	585 (90.7)	Ref		Ref	
Yes	130 (5.8)	70 (4.4)	60 (9.3)	2.23 (1.56–3.20)	<0.001	1.88 (1.09–3.22)	**.022**

CI = confidence interval, SD = standard deviation

Over a quarter of the sample (n = 600, 26.8%) was a member of one or more priority populations affected by bloodborne viruses. Over a quarter of the sample (27.6%) had a sexual identity other than heterosexual (see Table 1), including gay (10.3%), lesbian (2.6%), bisexual or pansexual (9.5%), and queer (5.4%) participants (noting that participants could identify with more than one sexual identity, e.g., gay and queer). Of the priority population groups targeted in recruitment, gay and bisexual men comprised 12.4% of the sample, people living with HIV 4.8%, sex workers 6.2% and trans and gender diverse people 5.8%. There were smaller numbers of participants from priority populations not specifically targeted in recruitment, including Aboriginal and/or Torres Strait Islander people (n = 67, 3.0%), people living with hepatitis B (n = 15, 0.7%) or C (n = 27, 1.2%), and people who injected drugs (n = 36, 1.6%). Because of the low frequencies, these groups were not included as covariates in the subsequent analyses.

### Knowledge of My Health Record

Of the whole sample (N = 2,240), 300 (13.4%) reported that they knew nothing about My Health Record, 1,295 (57.8%) knew a small or fair amount, and 645 (28.8%) knew quite a bit or a lot. The most common sources of knowledge of My Health Record among those who had heard of it (n = 1,940) were media coverage (49.0%), government advertising (44.2%), and conversations with friends and family (30.2%). For the analysis of the factors associated with knowledge of My Health Record, those who knew nothing, a small or fair amount were grouped together (less knowledge) and were compared with those who knew quite a bit or a lot (more knowledge; see Table 1). Greater knowledge of My Health Record was independently associated with younger age, having a university degree, having one or more health conditions, and being trans or gender diverse. Being a student, unemployed, or receiving government benefits, or having poor or fair self-reported health, were independently associated with less knowledge of My Health Record. Knowledge of My Health Record was not independently associated with the other sociodemographic variables or the other priority population groups.

### Accessing, opting out or deleting My Health Record

Of the whole sample (N = 2,240), 523 (23.3%) participants had accessed their My Health Record at least once, 577 (25.8%) had an active record but had never accessed it, 404 (18.0%) did not know if they had a record, and 78 participants (3.5%) were not eligible for one. These groups were combined and compared with the participants (n = 658, 29.4%) who had opted out or deleted their record. The analysis of factors associated with opting out or deleting My Health Record is shown in Table 2. The likelihood of opting out was independently associated with having a university degree, having one or more health conditions, being a gay or bisexual man, having another non-heterosexual identity, being HIV-positive, a sex worker, or trans or gender diverse. The likelihood of opting out was lower among people born overseas, residents of Queensland, and participants who were students, unemployed, or receiving government benefits. The likelihood of opting out was not independently related to the other sociodemographic variables.

**Table 2:** Factors associated with opting out of or deleting My Health Record among survey participants (N = 2,240).

	All	Retained record	Opted out	Odds ratio (95% CI)	*p* value	Adjusted odds ratio (95% CI)	*p* value
	N = 2,240 (%)	n = 1,582 (%)	n = 658 (%)				
Mean age (SD)	42.5 (16.1)	43.0 (16.7)	41.2 (14.4)	0.99 (0.99–1.00)	.021	1.00 (0.99–1.01)	.854
Gender							
Man/male	969 (43.3)	701 (44.3)	268 (40.7)	Ref		Ref	
Woman/female	1182 (52.8)	842 (53.2)	340 (51.7)	1.06 (0.87–1.28)	.57	1.23 (0.97–1.56)	.091
Non-binary	56 (2.5)	23 (1.5)	33 (5.0)	3.75 (2.16–6.51)	< .001	0.81 (0.35–1.88)	.629
Other/Prefer not to answer	33 (1.5)	16 (1.0)	17 (2.6)	2.78 (1.38–5.58)	.004	1.13 (0.48–2.65)	.781
Country of birth							
Australia	1699 (75.8)	1170 (74.0)	529 (80.4)	Ref		Ref	
Overseas	541 (24.2)	412 (26.0)	129 (19.6)	0.69 (0.55–0.87)	.001	0.63 (0.50–0.81)	**< .001**
Education level							
High school	602 (26.9)	478 (30.2)	124 (18.8)	Ref		Ref	
Trade certificate	584 (26.1)	430 (27.2)	154 (23.4)	1.38 (1.05–1.81)	.019	1.32 (1.00–1.76)	.053
University degree	1054 (47.1)	674 (42.6)	380 (57.8)	2.17 (1.72–2.75)	< .001	1.80 (1.37–2.35)	**< .001**
Residential location							
Capital city	1366 (61.0)	922 (58.3)	444 (67.5)	Ref		Ref	
Other city/regional, rural or remote area	874 (39.0)	660 (41.7)	214 (32.5)	0.67 (0.56–0.82)	< .001	0.84 (0.68–1.05)	.12
State or territory							
New South Wales	781 (34.9)	522 (33.0)	259 (39.4)	Ref		Ref	
Victoria	536 (23.9)	373 (23.6)	163 (24.8)	0.88 (0.70–1.12)	.293	0.97 (0.75–1.25)	.829
Queensland	391 (17.5)	303 (19.2)	88 (13.4)	0.59 (0.44–0.77)	< .001	0.69 (0.51–0.94)	**.018**
Other jurisdictions	532 (23.8)	384 (24.3)	148 (22.5)	0.78 (0.61–0.99)	.04	0.82 (0.63–1.07)	.141
Employment status							
Full-time/self-employed	967 (43.2)	631 (39.9)	336 (51.1)	Ref		Ref	
Part-time	309 (13.8)	211 (13.3)	98 (14.9)	0.87 (0.66–1.15)	.328	1.08 (0.79–1.48)	.613
Student/unemployed/other	964 (43.0)	740 (46.8)	224 (34.0)	0.57 (0.47–0.69)	< .001	0.69 (0.53–0.90)	**.006**
Income level (AUD)							
Less than $40,000	859 (38.3)	635 (40.1)	224 (34.0)	Ref		Ref	
$40,000−$79,999	650 (29.0)	472 (29.8)	178 (27.1)	1.07 (0.85–1.35)	.569	0.91 (0.70–1.19)	.506
$80,000−$120,000	376 (16.8)	238 (15.0)	138 (21.0)	1.64 (1.27–2.13)	< .001	1.23 (0.88–1.72)	.219
More than $120,000	188 (8.4)	116 (7.3)	72 (10.9)	1.76 (1.26–2.45)	.001	1.21 (0.79–1.84)	.375
Prefer not to say	167 (7.5)	121 (7.6)	46 (7.0)	1.08 (0.74–1.56)	.693	1.03 (0.71–1.51)	.86
Health conditions							
No reported conditions	1166 (52.1)	882 (55.8)	284 (43.2)	Ref		Ref	
One or more long-term health conditions	1074 (47.9)	700 (44.2)	374 (56.8)	1.66 (1.38–1.99)	< .001	1.41 (1.14–1.76)	**.002**
Self-assessed health status							
Good/very good/excellent	1730 (77.2)	1206 (76.2)	524 (79.6)	Ref			
Poor/fair	510 (22.8)	376 (23.8)	134 (20.4)	0.82 (0.66–1.02)	.081		
Receiving mental health care							
No	1602 (71.5)	1175 (74.3)	427 (64.9)	Ref		Ref	
Yes	638 (28.5)	407 (25.7)	231 (35.1)	1.56 (1.28–1.90)	< .001	0.96 (0.76–1.22)	.765
Sexual identity							
Heterosexual	1622 (72.4)	1250 (79.0)	372 (56.5)				
Gay and bisexual men	277 (12.4)	156 (9.9)	121 (18.4)	2.61 (2.00–3.39)	< .001	1.78 (1.26–2.51)	.001
Lesbian and bisexual women and other non-heterosexual participants	341 (15.2)	176 (11.1)	165 (25.1)	3.15 (2.47–4.01)	< .001	1.89 (1.40–2.55)	< .001
HIV status							
HIV-negative/Untested or unknown	2133 (95.2)	1534 (97.0)	599 (91.0)	Ref		Ref	
HIV-positive	107 (4.8)	48 (3.0)	59 (9.0)	3.15 (2.13–4.66)	< .001	2.25 (1.38–3.66)	**.001**
Sex worker							
No	2101 (93.8)	1521 (96.1)	580 (88.1)	Ref		Ref	
Yes	139 (6.2)	61 (3.9)	78 (11.9)	3.35 (2.37–4.75)	< .001	2.16 (1.45–3.21)	**< .001**
Trans or gender diverse							
No	2110 (94.2)	1531 (96.8)	579 (88.0)	Ref		Ref	
Yes	130 (5.8)	51 (3.2)	79 (12.0)	4.10 (2.84–5.90)	< .001	2.74 (1.57–4.80)	**< .001**

CI = confidence interval, SD = standard deviation

Participants who had retained their My Health Record (n = 1,100) were asked which types of information in the record were useful to them and the benefits of having a record. Participants indicated that the most useful information was test results (66.2%), medication history (58.7%), immunisation history (57.2%) and doctors’ notes (55.1%), while 11.8% indicated there was no useful information in their record. Participants who had retained a record indicated that the main benefits of having a record were that their medical history could be accessed by different healthcare providers (63.2%), the convenience in having information in one place (60.5%), and that their care had improved because their doctor had better information about their needs (48.1%), while 12.9% indicated that there were no benefits to having a record. There were no significant differences between priority population members (n = 224) and general population members (n = 876) in perceived usefulness and benefits of My Health Record, except that general population members perceived greater potential benefit in making medical decisions for someone they cared for (12.1% vs. 36.0%, *X*^2^(1, 1,100) = 14.0, *p* < .001).

Participants who had opted out or deleted their record (n = 658) were asked why they had done so. The most common reasons given were concerns about the government’s capacity to protect data privacy (79.3%), data being shared between government agencies without consent (67.3%), information being hacked or leaked (67.2%), records being used by the government in ways that might disadvantage them (62.6%), data being used for commercial purposes (57.4%), and not trusting that health professionals would treat them with respect (45.7%). In general, among those who had opted out or deleted their record, priority population members had higher levels of concern than general population members, particularly about health professionals treating them with respect (58.5% of priority populations vs. 41.5% of the general population, *X*^2^(1, 656) = 45.9, *p* < .001), or their data being used by the government in ways that might disadvantage them (54.1% of priority populations vs. 45.9% of the general population, *X*^2^(1, 656) = 44.2, *p* < .001). Priority population members were more likely to have opted out or deleted their record because their doctor told them they should (63.4% of priority populations vs. 36.6% of the general population, *X*^2^(1, 656) = 6.5, *p* = .011), or because another person or organisation told them to opt out (64.7% of priority populations vs. 35.3% of the general population, *X*^2^(1, 656) = 6.1, *p* = .013).

## Discussion

We assessed knowledge of Australia’s national electronic health record system, My Health Record, and the likelihood of opting out in a sample of the general population and priority populations affected by HIV and sexually transmissible infections. We found that most of the sample (71.2%) knew little about My Health Record. While most participants (70.6%) appeared to have an active record, less than a quarter of participants (23.3%) had accessed their record and a larger group (29.4%) had opted out or deleted their record. This suggests that the opt out policy for My Health Record has resulted in a lot of people holding active records, but few people knowing much about the system or actively using their record. This aligns with government data and previous research suggesting that most interactions with the system are by health providers, not members of the public [6,18]. Despite relatively limited interactions with the system, those who had retained their records indicated that the system could be useful, particularly in storing information about test results, medication and vaccination history, and doctors’ notes. They also nominated benefits such as healthcare providers having access to their medical history, convenience in having information in one place and potential improvements to medical care.

We hypothesised that populations affected by HIV and sexually transmissible infections would know more about My Health Record and would be more likely to have opted out or deleted their record, due to heightened concerns about privacy, security and the sharing or misuse of their information, and community-led debates about these risks and benefits [14,29–34]. Priority population members did not appear to know more about My Health Record than members of the general population, except for trans and gender diverse participants. It is not clear why trans and gender diverse people were more likely to know about My Health Record than other people. It is possible that trans people had heightened concerns about the record system, particularly the potential for inadvertent or inappropriate disclosure of their gender that might result in poorer health care [24,35,41], such as focusing on their gender rather than acute health conditions [42]. They may also have invested extra time in working out whether the record would be beneficial to them or not, particularly if they were seeing a number of providers, including for gender affirming care [29]. The other factors associated with greater knowledge of My Health Record included younger age, a higher education level and having one or more health conditions, which aligns with Australian and international research on health literacy and digital or e-health engagement [13,43,44]. Having a lower socioeconomic status is regarded as a barrier to digital health engagement [44,45], which aligns with our finding that students, unemployed people, and those receiving government benefits had less knowledge of My Health Record. People in our study with poor self-rated health knew less about My Health Record, suggesting that dealing with acute poor health (as opposed to a managed chronic condition) may be more pressing than engaging with systems like My Health Record.

We assessed factors associated with opting out or deleting of My Health Record and found that all the priority populations we targeted in recruitment (gay and bisexual men, people living with HIV, sex workers, and trans and gender diverse people) were more likely than other people to have opted out of the system, supporting our hypothesis. Priority population and general population members shared concerns about the capacity of the government to protect their information and the potential sharing or misuse of their health data without consent, as has been noted in previous research [10,12]. Priority population members had heightened concerns about health professionals not treating them with respect and the potential misuse of their health information [14]. They were also more likely to have opted out of My Health Record due to advice from medical professionals and other people or organisations, suggesting public and community-led debates about the benefits and risks of My Health Record had played a role in their decision [17,29–35]. We also found that non-heterosexual participants, more educated participants and people with ongoing health conditions were more likely to have opted out. The latter finding is of particular concern given that the system is supposed to improve coordinated and efficient care for people with chronic conditions [1,4,6]. Finally, we found that people born overseas, residents of Queensland, and students, unemployed people and those receiving government benefits were less likely to have opted out. Some of these groups also appeared to know less about My Health Record, suggesting that many had simply retained a record in the opt out period rather than being actively involved with the system. Overall, our findings indicate that some groups who might benefit from better coordinated care (due to their marginalised status or health conditions) were more likely to have opted out of My Health Record, suggesting that concerns or distrust in the system continue to hamper its potential.

Our study limitations include the cross-sectional design and its non-random sample. The sample we recruited was broadly representative of the Australian population in terms of state/territory of residence [7,37], and participation in employment [46]. We under-recruited people who were born overseas (30% of Australia’s population in 2020) [47], although this may also reflect the disruption to international travel and migration caused by COVID-19. The proportion of our sample reporting one or more chronic health conditions (48%) was similar to national samples (47%) [48], but the proportion who had received recent mental health care (29%) was higher than in random household samples (17%) [49]. This may reflect our deliberate overrepresentation of priority populations affected by HIV and sexually transmissible infections; some of these groups experience higher levels of mental health problems than the general population [25,27,50]. It could also be a result of the stress caused by COVID-19 [51].

Our analysis of engagement with My Health Record found low levels of knowledge of the system, and that populations affected by HIV and sexually transmissible infections, gender and sexual minorities, more educated people and people with chronic health conditions were more likely to have opted out of the system. Opting out was attributed to concerns about the government’s capacity to protect data in the system, data being shared between agencies without consent, and fears of hacking or leaks. This indicates ongoing problems with building trust in the system and providing meaningful safeguards to people who have concerns about the use of their personal health information. Our results suggest an ongoing need to engage with Australians about the utility and benefits of the My Health Record system. To build trust in the system, it may be necessary to consult priority populations about the design of the system, bolster its security and control features, demonstrate how the government has responded to previous problems with digital systems, and invest in sustained and meaningful community engagement. This may require additional investment in community-based education to support people who want to use electronic health records without compromising their health care, privacy, or security [11,16,36].

Prior to the rollout of My Health Record, state and territory-based trials (e.g. HealthConnect trials) demonstrated the possible benefits of electronic health records. These trials involved clinicians from different services explaining to patients how electronic records work and mutually agreeing the type and extent of information to be shared between providers. This was regarded as successful by governments and consumer advocates, particularly for patients seeing multiple specialists or those travelling long distances between services [52–54]. International research has also found that patients are more likely to use electronic health records if they are shown how to use them [55]. It is possible that a similar model could be used with the populations we worked with to increase the benefits and utility of My Health Record, with clinicians discussing with patients what information should be uploaded and with whom it should be shared. However, efforts like this would also need to address the concerns and fears of priority populations, as well as identify more explicitly the positive benefits of electronic health records.

The priority populations we worked with have understandable concerns about sharing personal or health information in a permanent system like My Health Record, and it is possible that further explanations of the system may not result in greater uptake or use, if the perceived benefits of the system do not outweigh its risks [56]. For example, trans and gender diverse people commonly experience discriminatory treatment in health care settings, and may be highly selective in where they seek care [24,57]. HIV remains a stigmatised condition, and people living with HIV may have understandable fears about their status being disclosed without their consent [26,58,59]. Lesbian, gay, bisexual and other non-heterosexual people may actively compartmentalise aspects of their health care, selectively disclosing details about their sexuality and sexual practices to some providers and not others to avoid judgmental treatment and discrimination [60–62]. Sex work remains subject to regulation and criminalisation in some Australian jurisdictions, and people may actively avoid disclosing sex work to health providers for that reason [34,63,64]. Involving these communities in discussions about My Health Record will not necessarily prevent discriminatory treatment or negative attitudes from providers. Choosing to opt out of a system such as My Health Record may therefore be a reasonable choice for participants who do not wish there to be a permanent record of their personal characteristics, health or employment status that may result in judgment, discrimination or poorer care.

It is possible that a system like My Health Record could adopt enhanced security measures so that sensitive information (like sexual orientation, HIV status or sex work) is automatically protected and cannot be shared without a patient’s consent. Education about these improvements could be targeted to the priority populations with whom we worked, particularly those who see multiple healthcare providers. Unfortunately, we believe there remains a bigger challenge to be addressed: explaining the value and utility of the My Health Record system to the majority of consumers who do not actively use it [6]. International reviews of electronic health record systems suggest that low levels of consumer engagement are the norm and implementing electronic health records does not necessarily lead to improved care, suggesting a mismatch between professional and consumer expectations of digital health systems [55,56]. Adopting technical enhancements may enhance user control and confidence, but they are unlikely to encourage greater participation in the system if patients perceive more risks than benefits in systems like My Health Record.

## Dryad DOI

10.5061/dryad.r2280gbgq [40]
